# Impaired Glucose Metabolism in Response to High Fat Diet in Female Mice Conceived by *In Vitro* Fertilization (IVF) or Ovarian Stimulation Alone

**DOI:** 10.1371/journal.pone.0113155

**Published:** 2014-11-18

**Authors:** Miaoxin Chen, Linda Wu, Fang Wu, Gary A. Wittert, Robert J. Norman, Rebecca L. Robker, Leonie K. Heilbronn

**Affiliations:** 1 Robinson Research Institute, School of Paediatrics and Reproductive Health, The University of Adelaide, Adelaide, Australia, 5005; 2 Department of Obstetrics and Gynaecology, The Affiliated Hospital of Guiyang Medical College, Guiyang, China, 550004; 3 Discipline of Medicine, The University of Adelaide, Adelaide, Australia, 5005; 4 Department of Emergency, The Affiliated Hospital of Guiyang Medical College, Guiyang, China, 550004; University of Alabama at Birmingham, United States of America

## Abstract

Individuals conceived by *in vitro* fertilization (IVF) may be at increased risk of cardio-metabolic disorders. We recently reported that IVF conceived male mice displayed impaired glucose metabolism at normal and high body weights. In this study, we examined glucose metabolism in mature female C57BL/6J mice that were conceived by natural conception (NC), by ovarian stimulation (OS) or by IVF following chow or high-fat diet (HFD) for 8 weeks. By design, litter size was comparable between groups, but interestingly the birth weight of IVF and OS females was lower than NC females (p≤0.001). Mature IVF female mice displayed increased fasting glucose as compared to NC and OS mice, irrespective of diet. Mature IVF and OS mice were also more susceptible to the metabolic consequences of high fat diet as compared with NC females, with impaired glucose tolerance (p≤0.01), whereas peripheral insulin resistance and increased hepatic expression of gluconeogenic genes *Ppargc1α, Pck1* and *G6pc* was observed in IVF mice only (p<0.05). This study suggests that ovarian stimulation alone and IVF program distinct metabolic effects in females, but that high fat diet may be required to unmask these effects. This study adds to the growing body of literature that assisted reproduction procedures may increase the risk of developing type 2 diabetes in an obesity prone environment.

## Introduction

Adverse prenatal environments are linked to the development of cardiovascular disease and type 2 diabetes in adult human, rodent and sheep offspring [1]–[4]. In particular, maternal low protein diets fed exclusively during the preimplantation period, resulted in hypertension in mature rats [5], [6] and mice [7], as well as cardiovascular dysfunction in mature sheep [8]. *In vitro* fertilization (IVF) exposes the preimplantation embryo to a non-physiological environment during embryo culture, and has been associated with changes in embryo growth and development in animal models and humans [9], [10]. An increased risk of metabolic and cardiovascular disease has also been shown in some cohorts of IVF children [11]–[14] and is documented in mouse models [15]–[17]. We have recently demonstrated insulin resistance in a small cohort of IVF-conceived young adult men and women, and also showed that these individuals were more susceptible to adverse outcomes of short-term high fat overfeeding versus naturally conceived controls [18]. Similarly, we observed that mature IVF male mice displayed impaired glucose tolerance, under chow and high fat fed conditions [18].

Evidence from humans and animal models shows that male embryos grow faster than female embryos [19], and metabolic differences are generally more profound in male than female offspring in response to an adverse periconceptional maternal environment [4], [20]. However, a previous mouse study [16] reported that IVF female offspring were glucose intolerant, but males maintained normal glucose tolerance, although both sexes displayed evidence of insulin resistance.

The process of IVF generally involves not only embryo culture, but ovarian hyperstimulation (OS), which itself has been associated with poorer perinatal outcomes, including low birth weight and preterm birth [21]–[23]. One study showed that 4-year-old children conceived following stimulated IVF had higher systolic blood pressure versus 4 year IVF children conceived following unstimulated or ‘natural IVF’ [24]. This study suggests the process of ovarian stimulation may be responsible for at least some of the phenotypes, however these differences may also have been related to the genetics or subfertility of the parent/s. In mature male mice, we recently showed that poor fetal growth could be attributed to ovarian stimulation since birth weight was lower in both OS and IVF groups, but that differences in glucose metabolism were evident solely in mice generated by IVF and not OS alone [18].

The aim of the current study was to examine whether mature female C57BL/6J mice conceived by IVF or by ovarian stimulation alone have differences in birth weights or glucose metabolism as compared with naturally conceived female mice following chow or high fat diet (HFD) for 8 weeks.

## Materials and Methods

### Mice

C57BL/6J male and female mice were obtained at 6 weeks of age, and vasectomized (CBA X C57BL/6) F1 male mice were obtained at 8 weeks of age from the Animal Resource Centre (Perth, Western Australia). (CBA X C57BL/6) F1 female mice were obtained at 6 weeks of age from the University of Adelaide Laboratory Animal Services (Adelaide, South Australia). All mice were maintained at 24°C on a 12 h light, 12 h dark cycle, with standard rodent chow diet with 20% of calories from fat, 16% of calories from protein, and 64% of calories from carbohydrate (SF06-105, Specialty Feeds, Glen Forrest, Australia) and water available ad libitum. All mice were acclimatised for 2 weeks on chow before experimentation. All experiments were approved by the University of Adelaide Animal Ethics Committee (Permit Number: M-2011-248) and were conducted in accordance with the Australian Code of Practice for the Care and Use of Animals for Scientific Purposes. All surgeries were performed under Avertin anesthesia, and all efforts were made to minimize suffering.

### Generating blastocysts

Mouse blastocysts were generated by natural conception (NC group), or by ovarian hormonal stimulation followed by either mating (OS group) or by IVF and embryo culture (IVF group) as reported [18]. For the NC group, female C57BL/6J mice were placed with male C57BL/6J mice overnight. For the OS group, female C57BL/6J mice were superovulated with intraperitoneal injections of 7.5 IU equine chorionic gonadotropin (Calbiochem, San Diego, CA) followed by 7.5 IU human chorionic gonadotropin (hCG; Calbiochem) 48 h later. Hormone dosages for hyperovulation in mouse strains range from 2.5 to 10 IU [25], and7.5 IU was selected in this study following testing for the optimal dose and as reported previously [26]. After injection with hCG, these females were placed with male C57BL/6J mice overnight. The following morning, female mice (NC and OS groups) with the presence of vaginal plugs were considered pregnant and were humanely sacrificed by cervical dislocation 3 days later. Blastocysts were collected by flushing dissected uteri with pre-warmed HEPES-buffered minimal essential medium (Invitrogen Australia Pty. Ltd.) supplemented with 5 mg/ml human serum albumin (SAGE Media ART-3001, CooperSurgical, USA). Blastocysts from the NC and the OS group were placed in Research Vitro Cleave Media (Cook Medical; QLD, Australia) for no more than 1 hour prior to being transferred to uteri of pseudopregnant mice (see below).

For the IVF group, the ovarian stimulation protocol was the same as for the OS group. *In vitro* fertilization was performed using a modified version of that described previously [27]. At 13 hours after injection with hCG, female C57BL/6J mice were humanely killed by cervical dislocation. Cumulus-oocyte complexes were placed in Research Vitro Fertilization Media (Cook Medical; QLD, Australia) under paraffin oil (Calbiochem) and incubated in a modular incubator chamber at 37°C in 6% CO_2_, 5% O_2_, 89% N_2_ for 5–6 hours with sperm, collected from the cauda epidydimis of male C57BL/6J mice, that had been previously incubated for 1 hour in Research Vitro Fertilization Media (Cook Medical; QLD, Australia) for capacitation. Putative zygotes were placed in Research Vitro Cleave Media (10 zygotes/20 µl drop; Cook Medical; QLD, Australia) and checked for fertilization/cleavage the next morning. Embryos (2-cell) were then transferred to a new drop of Research Vitro Cleave Media (Cook Medical; QLD, Australia) and cultured in the modular incubator chamber at 37°C in 6% CO_2_, 5% O_2_, 89% N_2_ for a further 2 days to the blastocyst stage (90–96 hours post-hCG injection).

### Blastocysts transfer

Sterility of vasectomized (CBA X C57BL/6) F1 males was confirmed by mating them three times with superovulated (CBA X C57BL/6) F1 females and verifying the absence of embryos in the reproductive tract three days later. To generate pseudopregnant recipient females for embryo transfer, unstimulated (CBA X C57BL/6) F1 female mice were mated with vasectomized (CBA X C57BL/6) F1 male mice and those with copulatory plugs the next morning were considered as day 0.5 of pseudopregnancy. At day 2.5 of pseudopregnancy, totally 7 to 10 blastocysts were transferred to uteri (3–5 blastocysts/horn) of each pseudopregnant mouse anesthetized by i.p. injection of Avertin (0.5 mg/g body weight, Sigma-Aldrich, St. Louis, MO). Analgesia Carprofen (5 mg/kg) (Rimadyl Pfizer) was injected subcutaneously once after the surgery.

### Pups and diets

Pups were born on day 19.5 of pregnancy and birth weights measured the next morning. Body weights of pups were measured weekly. Growth rate was calculated as described previously [28]. Pups were weaned at 3 weeks of age onto chow diet or high-fat diet (HFD) for 8 weeks. High-fat diet was made in house using the same recipe as D12492 of Research Diets (New Brunswick, NJ); 60% energy from fat with 91% lard and 9% soybean oil, 20% energy from protein, and 20% energy from carbohydrate. Only one or two female offspring were taken from each litter and were examined for separate tests below in this study.

### Glucose and insulin tolerance tests

At 11 weeks of age, mice (n = 6–8) were fasted for 6 hours (from 8 am to 2 pm) and were then challenged with either an intraperitoneal injection of glucose (2 g/kg) or insulin (Actrapid, 0.75 U/kg, Novo Nordisk Australasia) for glucose or insulin tolerance tests respectively. Blood samples were obtained from the tail tip for assessment of glucose at 0, 15, 30, 60, 120 minutes with a glucometer (AccuChek Performa Monitor, Roche Diagnostics, Indianapolis, USA), and for insulin assays at 0, 30, 60, 120 minutes during glucose tests only by ultra-sensitive ELISA kits (Millipore). At 12 weeks of age, mice were sacrificed by cervical dislocation; inguinal fat, parametrial fat, quadriceps and liver were immediately excised and snap-frozen. Tissue samples were frozen at −80°C for later assessment.

### Quantitative real-time PCR

Total RNA (n = 6) was extracted from liver and quadriceps using Trizol (Invitrogen, USA) following manufacturer’s instructions. The concentration and purity of RNA were determined by Nanodrop (Thermo Fisher Scientific, California, USA). cDNA was synthesized from 1 µg of each RNA sample in 20 µl reactions using the QuantiTect reverse transcription kit (Qiagen, Valencia, CA). Standard control samples (25 ng/µl) pooled from each cDNA sample were diluted to create a standard curve as described previously [29].

Quantitative real-time PCR was performed as described previously [29] in duplicate with the ABI 7500 sequence detection system (Applied Biosystems, Foster City, CA) using TaqMan Fast Universal Master Mix and TaqMan primers and probes listed in Table S1. These genes are key regulators of mitochondrial biogenesis *Ppargc1α* (peroxisome proliferator-activated receptor gamma, coactivator 1 alpha), *Ndufb5* (NADH dehydrogenase (ubiquinone) 1 beta subcomplex, 5) and *Tfam* (Mitochondrial transcription factor A), lipid metabolism *Srebf1* (Sterol regulatory element-binding transcription factor 1) and *Cpt1a* (carnitine palmitoyltransferase 1A), and glucose metabolism *Gck* (Glucokinase), *G6pc* (Glucose-6-phosphatase catalytic subunit) and *Pck1* (phosphoenolpyruvate carboxykinase 1, cytosolic). The NormFinder program was used as described previously [30] and *Hprt* (Hypoxanthine phosphoribosyltransferase) and *Ppia* (Cyclophilin-A) out of 7 potential reference genes were identified as the best combination of reference genes. Data were analyzed using the 2^−(ΔΔCT)^ method and expressed as the fold change relative to a control sample, which was selected from the NC group fed on chow and was included in each run.

### Western immunoblotting

Liver and quadriceps (n = 4) were lysed and protein concentration was determined by bicinchoninic acid assay (Pierce BCA Protein Assay Kit, Thermo Scientific). Lysates (20 µg protein) were resolved by SDS-PAGE (Criterion XT 10% Bis Tris precast gels, Bio-Rad, Australia) and transferred onto PVDF membranes (criterion gel blotting sandwiches, Bio-Rad, Australia). Membranes were blocked in Tris-buffered saline with Tween 20 (TBST) [10 mM Tris, 150 Mm NaCl, and 0.1% Tween 20 (pH 7.5)] containing 5% (wt/vol) Amersham ECL blocking agent (GE Healthcare Australia Pty. Ltd., NSW, Australia) for 1 hour at room temperature. Membranes were then incubated with primary antibodies overnight at 4°C in 5% bovine serum albumin/TBST. Primary antibodies used were PPARGC1A antibody at 1∶1000 (Abcam, MA, USA, Cat. No ab54481) and Total OXPHOS (oxidative phosphorylation) antibody cocktail at 1∶1000 (Abcam, Cat. No ab110413). Beta-tubulin antibody at 1∶1000 (Cell Signaling, Cat. No 2146) was used as reference protein. Membranes were then washed in TBST and incubated with sheep anti-rabbit IgG alkaline phosphatase conjugated secondary antibody at 1∶2500 (Millipore, Temecula, CA) or goat anti-mouse IgG alkaline phosphatase conjugated secondary antibody at 1∶2000 (Millipore, Temecula, CA) for 1 hour. ECF substrate (GE Healthcare) was prepared and applied to the blots and then all blots were scanned for fluorescence by the Typhoon Trio^+^ (GE Healthcare) following the manufacturer’s instructions. The band intensity was measured using Image J software (The National Institutes of Health, USA).

### Mitochondrial DNA (mtDNA) copy number quantification

DNA were extracted from quadriceps (10 mg) using a QIAamp DNA Micro Kit (Qiagen) following manufacturer’s instructions. The concentration and purity of DNA were determined by Nanodrop (Thermo Fisher Scientific). DNA was then used to estimate average mtDNA copy number/cell as described in [31]. The primers for amplification of the mitochondrial gene (12S rRNA) were 5′-CGT TAG GTC AAG GTG TAG CC-3′ and 5′-CCA GAC ACA CTT TCC AGT ATG-3′. The primers for amplification of the nuclear gene (β-actin) were 5′-GGAAAAGAGCCTCAGGGCAT-3′ and 5′-CTGCCTGACGGCCAGG-3′. PCR amplification of mitochondrial DNA and nuclear DNA was performed simultaneously in each sample in triplicate using SYBR Green PCR Master Mix (Applied Biosystems) and a Rotor-Gene 6000 (Corbett, Valencia, CA) real-time rotary analyzer. The Ct value for β-actin was subtracted from that for 12S rRNA to give the ΔCt value and mtDNA copy number per nuclear genome (two actin gene copies) is calculated as 2×2^−(ΔCt)^.

### Statistical analysis

Data are shown as mean ± the standard error of the mean, unless otherwise stated. Data were analyzed statistically with SPSS 20 (SPSS, Chicago, IL, USA) and log-transformed for analysis if not normally distributed. Area under the curve(AUC) for glucose and insulin was calculated using the trapezoidal rule [32]. Total OXPHOS protein levels were calculated as the mean of OXPHOS complexes I–V and the relative density of PPARGC1A and total OXPHOS was normalized by beta-tubulin. Single comparisons were performed with one-way ANOVA for treatment effect or two-way ANOVA with diet and treatment as between group factors, whereas time-courses were analyzed by repeated-measures ANOVA and Bonferroni post hoc analysis. Insulin sensitivity assessed by insulin tolerance test is represented by delta glucose levels between baseline (0 minute) and 15 minutes and analyzed by two-way ANOVA, adjusting for baseline glucose levels which were significantly different between groups and were related to the change in glucose. Nonparametric tests (Mann-Whitney U test or Kruskal-Wallis test) were used when required. Chi square tests were used for nominal data. Differences were considered statistically significant at P<0.05.

## Results

### Reduced fetal growth and postnatal catch-up growth in OS and IVF mice

**Figure 1:**
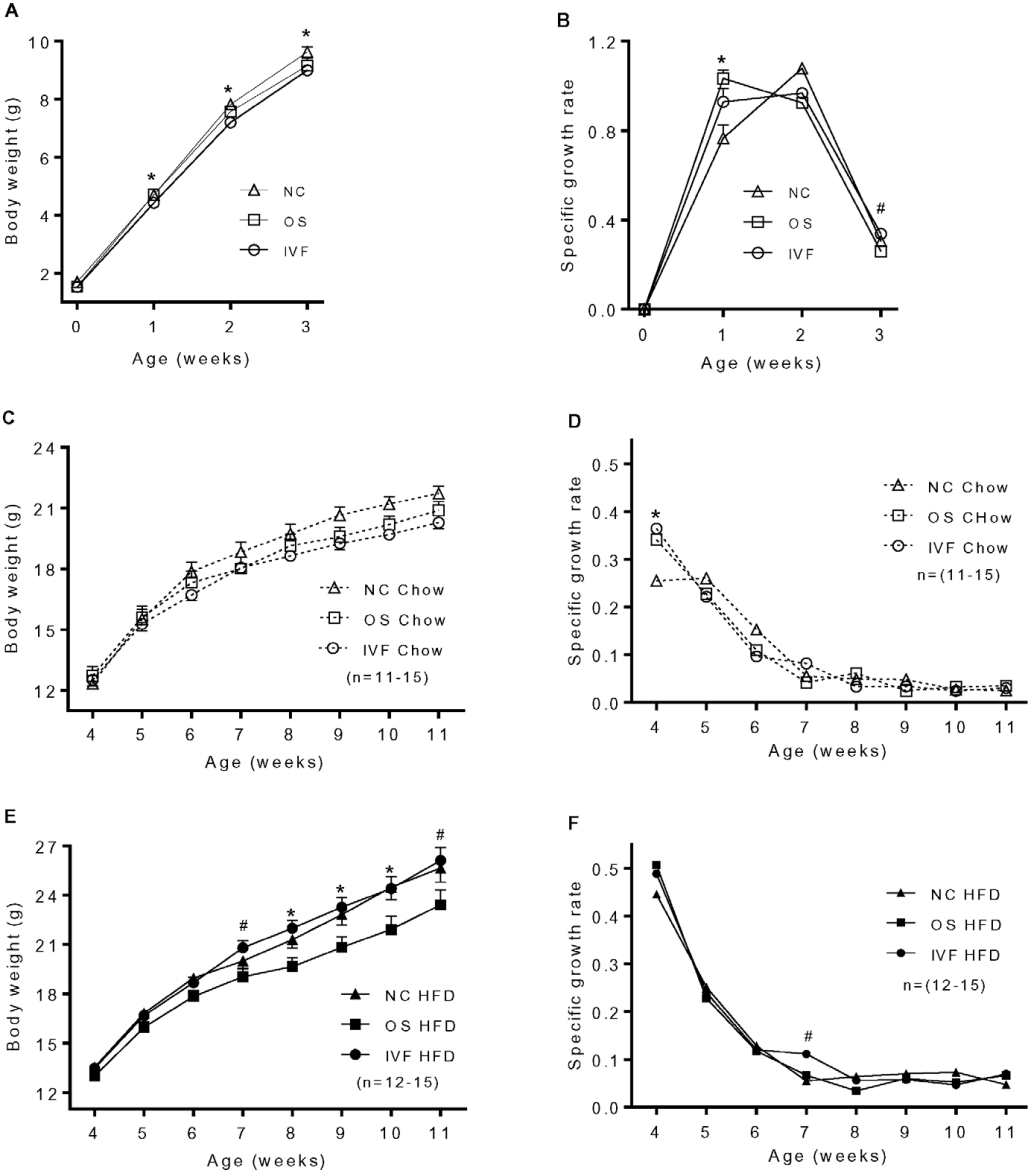
Body weight and growth rate in female mice offspring. Specific growth rate was calculated as follow: (Wn−W(n−1)**/**W(n−1). W is body weight, and n is age in weeks. (A), *IVF vs NC, P = 0.04; (n = 21**–**26). (B), *OS vs NC, P = 0.004; #IVF vs OS, P = 0.006. (n = 21**–**26). (D), Chow: *IVF vs NC, P = 0.03. (E), HFD: #IVF vs OS, P<0.001; *IVF & NC vs OS; P<0.01. (F), HFD: #IVF vs OS & NC, P<0.01.

Litter size and sex ratio were not different between groups (Both P = 0.5, Table 1). Despite this, birth weight was significantly lower in the IVF and OS versus the NC female pups (P≤0.001, Table 1). This difference in body weight was maintained in the IVF female pups until weaning (P = 0.04, Figure 1A), whereas the OS pups displayed rapid catch-up growth as shown by increased growth rate as compared with the NC pups at 1 week (P = 0.004; Figure 1 A,B). After weaning, a significantly increased growth rate was observed at 4 weeks of age in IVF vs NC females fed a chow diet and at 8 weeks of age in IVF vs NC and OS females fed a HFD (P<0.04, Figure1 D,F). OS mice weighed significantly less than IVF and NC mice from 4 weeks after exposure to HFD (P<0.01, Figure 1E). In a subset of animals (n = 4/group), liver weight and adipose tissue fat pads were weighed. As expected, the liver weight ratio was decreased and adipose tissue weight ratio increased in mice that were fed a HFD (P<0.001, Figure S1). However, a significantly higher percentage of body weight was in the inguinal and parametrial fat pads in OS and IVF versus NC mice fed HFD (P≤0.001, Figure S1).

**Table 1:** Litter characteristics and birth weight of female pups.

Group	Pups	Litters	Litter size	Range of litter size	Sex ratio(M/F)	Females	Birth weight(g)
NC	49	8	6.2± 0.6	4–8	1∶1.33	28	1.69 ± 0.04
OS	51	7	7.4± 0.7	5–10	1∶0.82	23	1.54 ± 0.03Δ
IVF	54	8	6.9± 0.5	5–9	1∶1.08	28	1.52 ± 0.03*

NC: natural conception; OS: ovarian stimulation; IVF: in vitro fertilization. Data presented as mean ± SEM; Litter size (P = 0.5); M/F: male/female (P = 0.5); The litter is used for comparisons of birth weight;

Δ OS vs NC, P = 0.001,

*IVF vs NC, P<0.001.

### Impaired glucose metabolism in OS and IVF female mice following HFD

**Figure 2:**
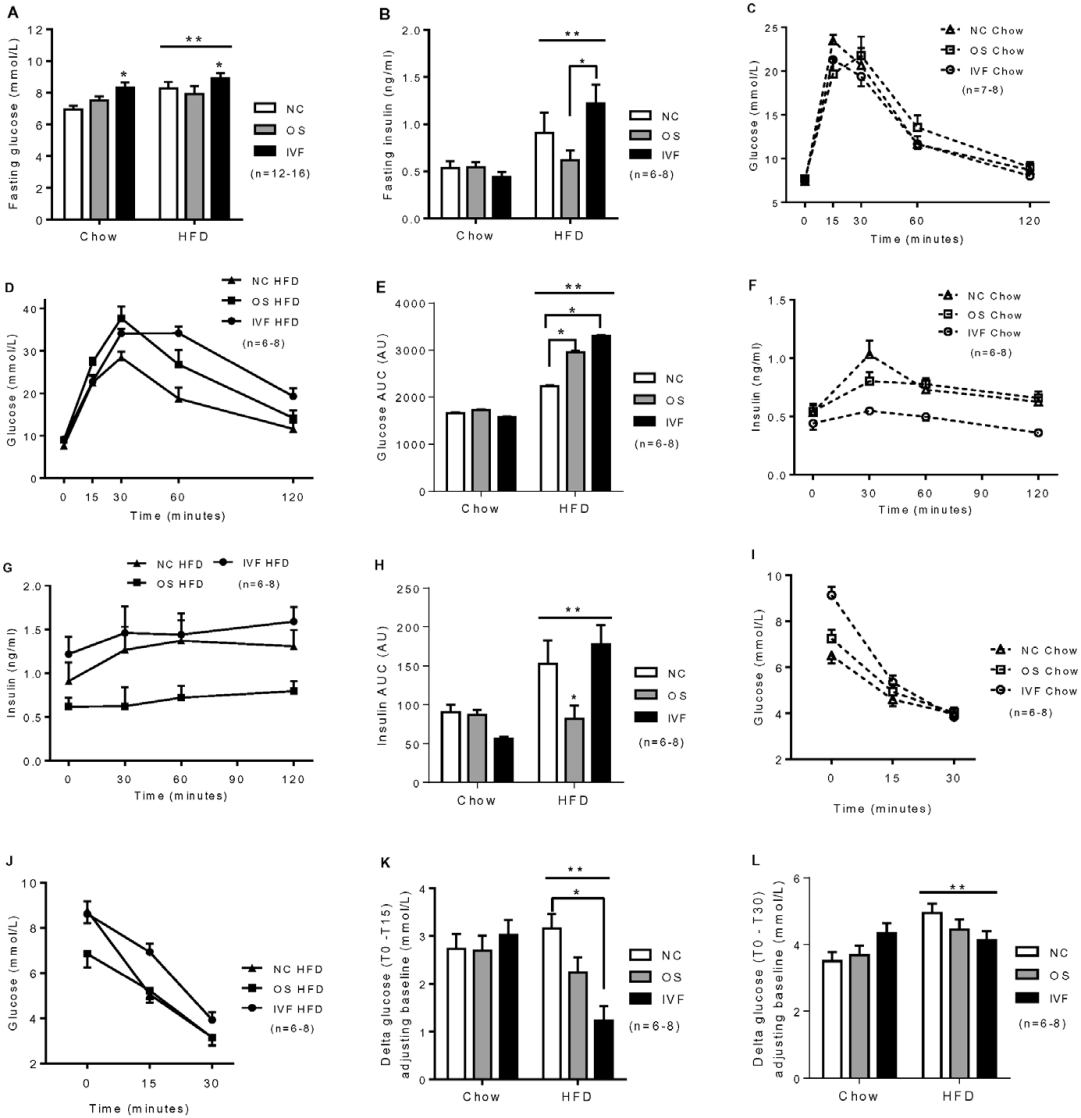
Fasting glucose, fasting insulin, glucose (C–E) and insulin (F–H) response to intraperitoneal glucose tolerance tests, and insulin tolerance tests (I–K) in female mice offspring. AU, arbitrary unit. (A), *IVF vs NC, P = 0.01; IVF vs OS, P = 0.03; **Diet effect, P = 0.01. (B), *IVF vs OS, P = 0.03; **Diet effect, P = 0.002. (E), *OS vs NC, P = 0.01; IVF vs NC, P<0.001; **Diet effect, P<0.001. (H), *OS vs IVF, P = 0.001,OS vs NC, P = 0.04; **Diet effect, P<0.001. (I–J), The glucose levels at T0 and T30 following insulin i.p. (K), Insulin sensitivity is represented by delta glucose adjusting baseline (T0–T15);*IVF vs NC, P<0.001; **Diet effect, P = 0.02. (L), Insulin sensitivity is represented by delta glucose adjusting baseline (T0–T30); **Diet effect, P = 0.01.

Fasting glucose was significantly higher in IVF versus NC and OS mice, independently of diet (P<0.05, Figure 2A). However, no other different phenotypes were noted in IVF or OS females versus NC females fed a chow diet. HFD increased fasting glucose, fasting insulin, glucose area under curve (AUC), insulin AUC and reduced insulin sensitivity (diet effect, P<0.05, Figure 2) and OS and IVF mice were more susceptible to adverse effects of HFD as evidenced by impaired glucose tolerance versus NC mice (P≤0.01, Figure 2E). Insulin sensitivity as assessed by insulin tolerance test was also significantly lower in IVF mice versus NC mice only at 15 minutes (P<0.001, Figure 2K). Lower fasting insulin (P = 0.03, Figure 2B) and lower insulin AUC was observed in OS mice as compared with NC and IVF mice in response to glucose tolerance test (P<0.05, Figure 2H).

**Figure 3:**
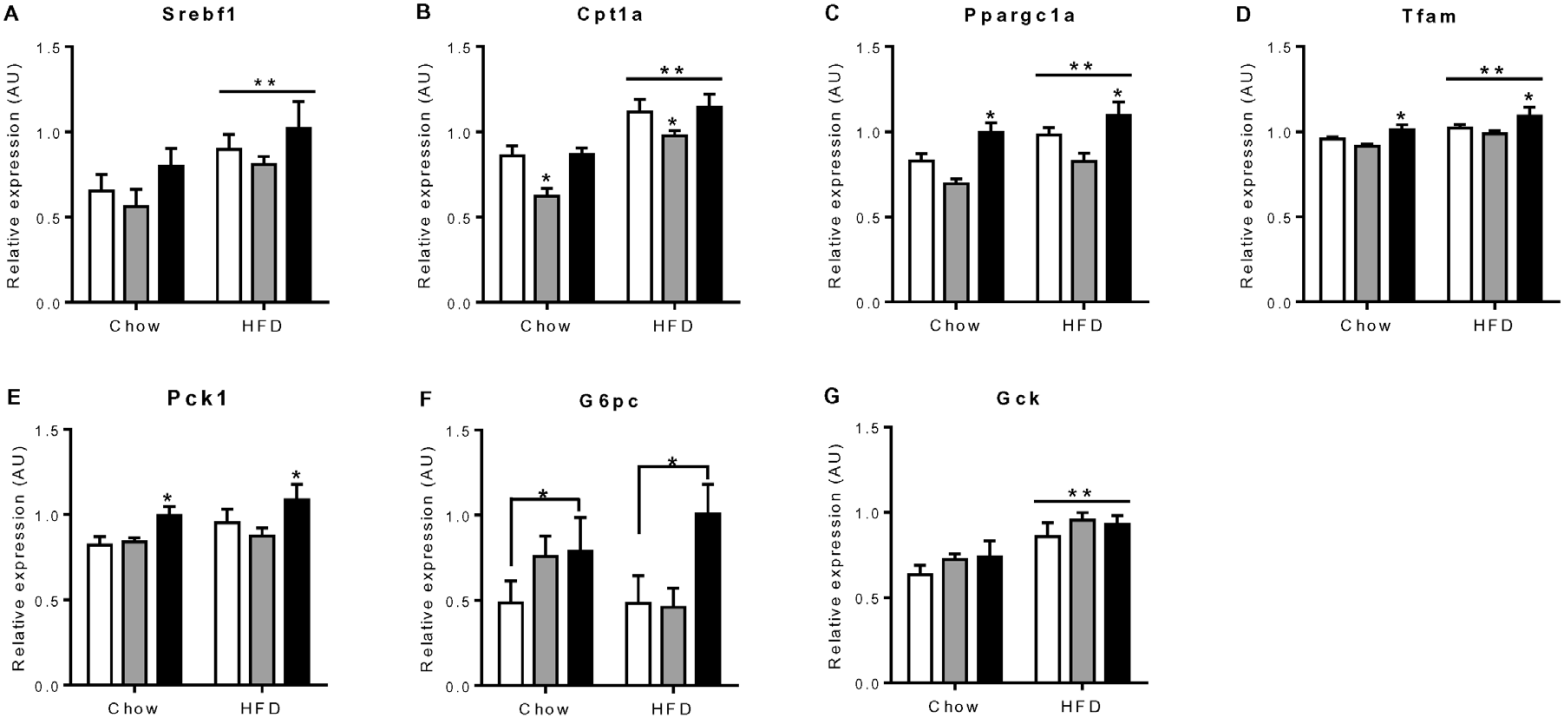
Hepatic gene expression of female mice offspring. White bars represent the NC group, grey bars represent the OS group and black bars represent the IVF group (n = 6). (A), **Diet effect, P = 0.009. (B), *OS vs IVF & NC, P<0.01; **Diet effect: P<0.001. (C), *IVF vs NC, P = 0.03; IVF vs OS, P<0.001; **Diet effect, P = 0.005. (D), *IVF vs OS, P = 0.003; **Diet effect, P = 0.003. (E), *IVF vs NC, P = 0.05; IVF vs OS, P = 0.02. (F), *IVF vs NC, P = 0.03. (G), **Diet effect, P<0.001.

**Figure 4:**
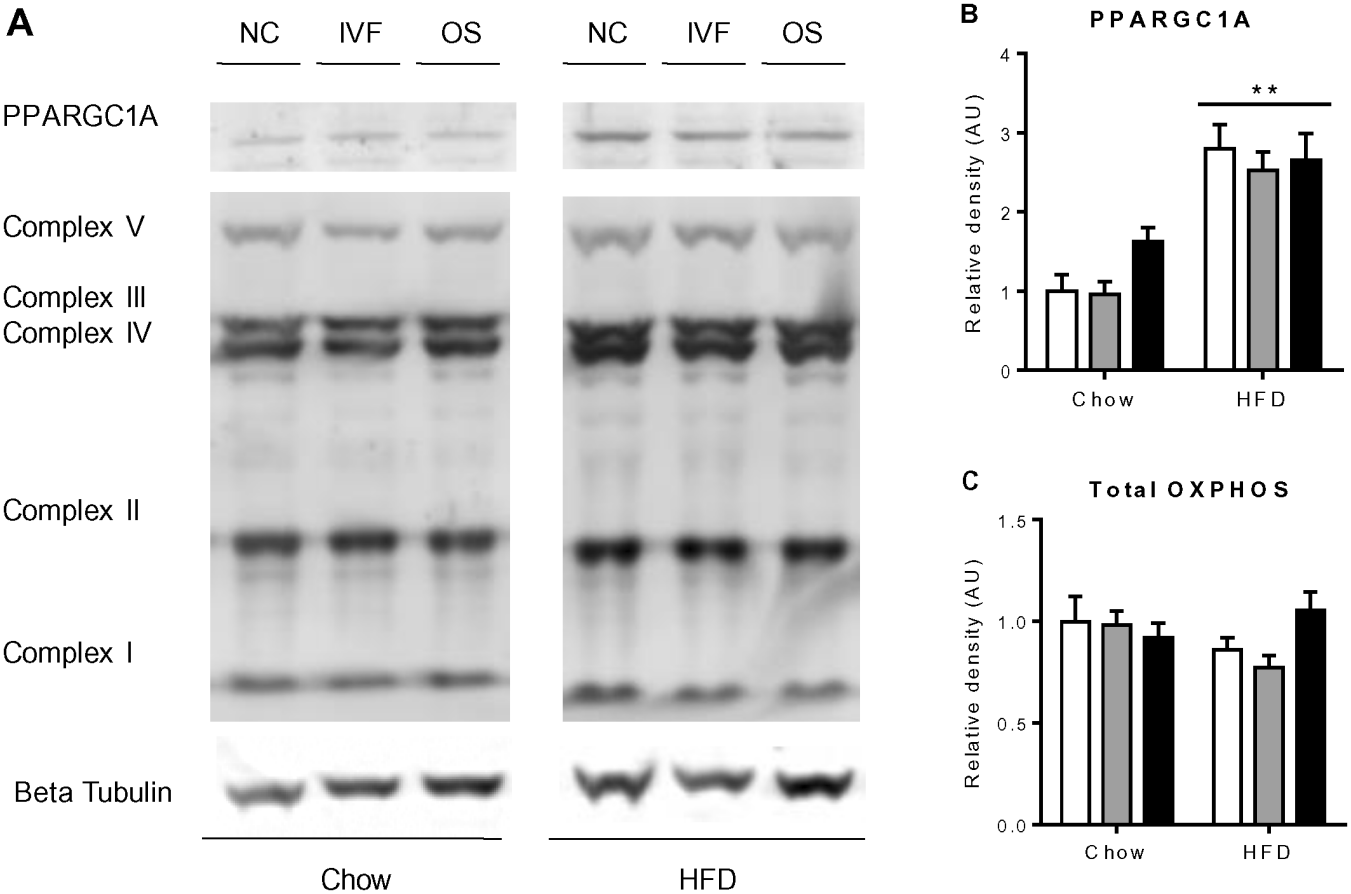
Protein levels of mitochondrial biogenesis markers PPARGC1A and total oxidative phosphorylation (OXPHOS) in liver of female mice offspring. White bars represent NC group, grey bars represent OS group and black bars represent IVF group (n = 4). (A), representative blots of PPARGC1A, OXPHOS complexes I–V and beta-tubulin. (B), **Diet effect, P<0.001.

We also examined the mRNA expression of genes involved in glucose and lipid metabolism in liver. HFD increased the expression of the genes *Srebf1, Cpt1a, Ppargc1α, Tfam,* and *Gck*, but not gluconeogenic genes *Pck1* and *G6pc* (Figure 3). Significant differences were also noted between groups, with increased hepatic expression of *Ppargc1α* and *Pck1* in IVF mice and decreased expression of *Cpt1a* in OS mice, independently of diet. The expression of *G6pc* and *Tfam* was also higher in IVF versus NC or OS mice respectively (Figure 3). However, no difference was observed in protein levels of mitochondrial biogenesis markers PPARGC1A and total OXPHOS in liver (Figure 4). These genes were also assessed in quadriceps muscle from animals fed HFD. No difference was observed in either gene expression or mitochondrial DNA copy number or protein levels of these makers in quadriceps muscle (data not shown).

## Discussion

Children conceived by IVF have some evidence of altered cardio-metabolic outcomes [14], [33]. We recently showed that IVF young adult humans displayed peripheral insulin resistance [18], an early risk factor in the development of type 2 diabetes [34], [35], and significantly greater increases in systolic blood pressure when exposed to a high fat overfeeding diet for 3 days [18]. Furthermore, we showed that male C57BL6/J mice conceived by IVF displayed hyperglycemia and impaired glucose tolerance when fed chow or HFD [18]. In this study, we now report that female mice conceived by IVF also exhibited higher fasting glucose levels, independently of diet. However, IVF female mice fed a chow diet did not display any other adverse risk factors, whereas impaired glucose tolerance was unmasked by HFD, but this was apparent in both IVF and OS females. This study suggests that manipulation of the preimplantation embryo may predispose mature female mice to increased risk of developing type 2 diabetes, in an obesogenic environment.

In this study, birth weight was lower in female mice conceived either by ovarian stimulation alone or by IVF. This is consistent with the results we have recently reported in male mice [18]. Lower fetal weights have also been reported in mice conceived following ovarian stimulation at day 14 and day 18 of gestation [36], [37]. Lower birth weights are also reported in human infants conceived by ovarian stimulation alone or by IVF after either fresh or frozen embryo transfer as compared with naturally conceived infants [21], [22], [38]–[43], although fresh embryo transfer further increases the risk of low birth weight and preterm birth in human infants as compared with frozen-thaw embryo transfer cycles [40], [42], [43]. In this study, while female donor mice for both the IVF and OS groups underwent ovarian hyperstimulation, the blastocysts for the OS group were developed in a hormonally stimulated reproductive tract prior to transfer, whereas the IVF blastocysts were developed *in vitro* in culture media prior to transfer. Previous studies have shown that both stimulated oviductal and *in vitro* culture environments are suboptimal and can impair embryo and fetal development in mice [44], [45].The mechanism underlying the association between ovarian stimulation and low birth weight was not a focus of this study. However, since the embryos were transferred to unstimulated recipients in this study, this suggests that differences may be due to impaired oocyte quality and/or subsequent embryonic and fetal development, rather than changes in endometrial receptivity [36], [37], [44], [46]. Further study is needed to determine if lower doses of gonadotropin result in less severe phenotypes in C57BL/6J mice and whether the OS phenotype is altered if kept in the stimulated uterine environment rather than undergoing embryo transfer.

Accelerated postnatal catch-up growth in infancy and early childhood are independent risk factors for cardiovascular disease and type 2 diabetes in humans [1], [2], [47]–[51] and rodents [52]–[54]. Accelerated weight gain in late infancy has been reported in IVF children as compared with spontaneously conceived children born to subfertile parents, and early childhood growth was also related to increased blood pressure in IVF children [55]. In this study, both OS and IVF female pups displayed catch-up growth. Of note, catch-up growth was observed in OS females soon after birth but occurred much later in IVF females after weaning, and OS mice on HFD failed to maintain this increase after 4 weeks on HFD. Sequential assessments of growth axis factors such as growth hormone, IGF, and ghrelin are needed during the early growth phase to tease out potential mechanisms underlying these differences in weight gain. Interestingly, and unlike our findings in males [18], both OS and IVF females fed on HFD displayed relatively higher adiposity, although this was only examined in 4 animals per group and thus should be confirmed with greater numbers and by other assessments of body composition. Further, assessments of food intake and energy metabolism should also be included in future studies to determine mechanisms underlying this difference in energy needs between groups. In this study, we also observed that IVF and OS mice displayed impaired glucose tolerance following HFD. Scott et al. also reported impaired glucose tolerance, and a compensatory insulin response to glucose in IVF versus naturally conceived B6C3F1 female mice at 8 weeks of age, even in response to chow diet [16]. Two more recent studies reported no differences in glucose tolerance between chow fed CF1xB6D2F1 or chow and HFD fed C57BL/6J female mice conceived by ovarian stimulation alone versus IVF [56], [57], whereas glucose intolerance was observed in IVF females when embryos were cultured in more stressful conditions as compared with OS females, but notably these studies did not test relative to an unstimulated naturally conceived control mouse, as was performed in this study by including the NC group.

Interestingly, the pathways underlying these differences in glucose tolerance may be distinct between OS and IVF groups. In OS females, impaired glucose tolerance was observed in conjunction with lower insulin, possibly indicating impaired β-cell function. However, we did not collect the pancreas, and thus cannot assess islet numbers or size, nor investigate the insulin response to IV or oral GTT. In IVF females fed a HFD, peripheral insulin resistance as assessed by insulin tolerance test was noted and likely responsible for the impaired glucose tolerance. These data supports our previous findings of peripheral insulin resistance in IVF young adults, of whom 70% were women, and in this group we did not detect any differences in β-cell secretory capacity in response to intravenous injection of glucose [18].

We recently showed that only IVF, and not OS, conceived male mice displayed impaired glucose intolerance versus NC, and that this was observed following either chow or HFD [18]. This difference between sexes indicates that sexual dimorphism may exist in offspring conceived by IVF. Sex differences in phenotypes have been identified in several developmental programming models of adult diseases both in humans and animals [4], [20]. Some of these differences may be because males grow faster than females in utero and thus are more vulnerable to malnutrition [58]. Furthermore, marked differences in the gene expression patterns are noted in male and female preimplantation embryos [59].

Uniquely, higher fasting blood glucose was observed in IVF females on chow and high fat diet, which we also observed in IVF males generated from the same litters [18]. We speculate this may be due to increased hepatic gluconeogenesis, since we observed increased expression of key gluconeogenic genes including *Ppargc1α, Pck1* and *G6pc* in the livers of IVF females. *Ppargc1α*is a key regulator of hepatic gluconeogenesis that contributes to circulating hyperglycemia [60], and liver-specific *Ppargc1α*-deficient mice have reduced *Pck1* and *G6pc* expression and mild hypoglycaemia [61]. IVF male mice displayed increased hepatic expression of key lipogenic gene *Srebf1* and impaired hepatic insulin sensitivity as evidenced by reduced AKT phosphorylation in liver following an insulin stimulation test [18]. Thus, whilst male and female IVF offspring both display hyperglycemia, this may be due to alternative activation of *Ppargc1α* or *Srebf1* pathway[62]. However, this result requires further testing. Importantly, our findings support previous results in humans that have shown higher fasting glucose levels in pubertal IVF children that could not be explained by environmental factors, including parental subfertility [12]. Taken together, the data suggests that *in vitro* culture induces profound alterations of metabolic regulatory pathways compared to the in vivo hormonally stimulated tract environment.

To our knowledge, only three studies have examined the long term effects of IVF on glucose metabolism in mouse models to date [16], [56], [57]. However, one group did not control for litter size or genetics between IVF and control groups, which may have impacted the outcomes. Two more recent studies compared IVF to models of ovarian stimulation alone [56], [57], which we and others have shown impairs fetal growth, and may also alter metabolic outcomes [36], [37], [44], [46], and one study utilized outbred mice, which are less suited for metabolic studies [57]. In this study, we performed blastocyst transfer in all three groups so that we could control for any effects of embryo transfer, and more importantly enabled matching of litter size, as well as maternal environment across all three groups.

In conclusion, ovarian stimulation reduced fetal growth in utero, and when coupled with accelerated catch up growth and HFD, results in adiposity and impaired glucose tolerance. Further, *in vitro* culture of embryos uniquely increased fasting glucose and markers of hepatic gluconeogenesis, suggesting that IVF in particular, may increase the risk of developing type 2 diabetes in adulthood.

## Supporting Information

Figure S1 **Tissue weight ratio (normalized to body weight) in female mice offspring.** **Diet effect, P<0.001. (A) Liver; (B), Inguinal fat, *OS vs NC, P = 0.02; IVF vs NC, P = 0.001; (C), Parametrial fat, *OS vs NC, P = 0.01; IVF vs NC, P<0.001.
(TIF)

Table S1 **TaqMan primers and probes used for gene expression analysis.**
(DOCX)
