# Supplementary figures and images for: Impaired Glucose Metabolism in Response to High Fat Diet in Female Mice Conceived by In Vitro Fertilization (IVF) or Ovarian Stimulation Alone
Source: PLoS One. 2014 Nov 18;9(11):e113155. doi: 10.1371/journal.pone.0113155 (PMC4236136; doi:10.1371/journal.pone.0113155)

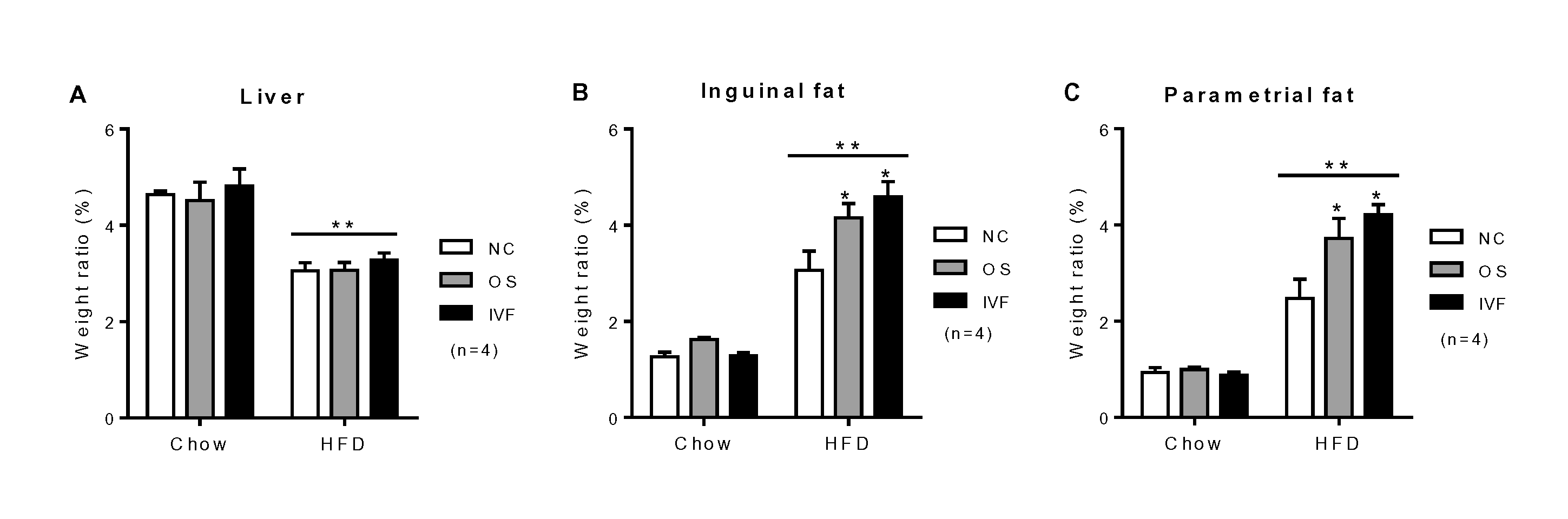

Supplement: Figure S1 — Tissue weight ratio (normalized to body weight) in female mice offspring. **Diet effect, P<0.001. (A) Liver; (B), Inguinal fat, *OS vs NC, P = 0.02; IVF vs NC, P = 0.001; (C), Parametrial fat, *OS vs NC, P = 0.01; IVF vs NC, P<0.001. (TIF) [file pone.0113155.s001.tif]
