# Supplementary material for: Impaired Glucose Metabolism in Response to High Fat Diet in Female Mice Conceived by In Vitro Fertilization (IVF) or Ovarian Stimulation Alone
Source: PLoS One. 2014 Nov 18;9(11):e113155. doi: 10.1371/journal.pone.0113155 (PMC4236136; doi:10.1371/journal.pone.0113155)
Supplement: Table S1 — TaqMan primers and probes used for gene expression analysis. (DOCX) [file pone.0113155.s002.docx]

Table S1 TaqMan primers and probes used for gene expression analysis

| Gene Symbol | Gene Name | Reference number |
| --- | --- | --- |
| Ppargc1a | Peroxisome proliferator-activated receptor gamma, coactivator 1 alpha | Mm01208835_m1 |
| Tfam | Mitochondrial transcription factor A | Mm00447485_m1 |
| Ndufb5 | NADH dehydrogenase (ubiquinone) 1 beta subcomplex, 5 | Mm00452592_m1 |
| Cpt1a | Carnitine palmitoyltransferase 1A | Mm01231183_m1 |
| G6pc | Glucose-6-phosphatase catalytic subunit | Mm00839363_m1 |
| Gck | Glucokinase | Mm00439129_m1 |
| Pck1 | Phosphoenolpyruvate carboxykinase 1, cytosolic | Mm01247058_m1 |
| Srebf1 | Sterol regulatory element-binding transcription factor 1 | Mm00550338_m1 |
| Gapdh | Glyceraldehyde-3-phosphate dehydrogenase | Mm99999915_g1 |
| Rn18s | 18S ribosomal RNA | Mm03928990_g1 |
| Hprt | Hypoxanthine phosphoribosyltransferase | Mm01545399_m1 |
| Ppia | Cyclophilin-A, | Mm02342430_g1 |
| B2M | Beta-2 microglobulin | Mm00437762_m1 |
| Actb | Beta actin | Mm00607939_s1 |
| Rplp0 | Ribosomal protein, large, P0 | Mm00725448_s1 |

All TaqMan primers and probes are from Life Technologies Australia Pty Ltd, VIC, Australia.
